# Traditional Chinese Medicine Tongxinluo Improves Cardiac Function of Rats with Dilated Cardiomyopathy

**DOI:** 10.1155/2014/323870

**Published:** 2014-12-28

**Authors:** Fang-Fang Shen, Ting-Hui Jiang, Jin-Qi Jiang, Ying Lou, Xu-Min Hou

**Affiliations:** ^1^Department of Emergency, Shanghai Chest Hospital, Shanghai Jiao Tong University, Shanghai 200030, China; ^2^Department of Integrated Traditional Chinese and Western Medicine, Shanghai Tong Ren Hospital, Shanghai 200050, China; ^3^Department of Cardiology, Shanghai Chest Hospital, Shanghai Jiao Tong University, Shanghai 200030, China

## Abstract

The study aimed at testing the hypothesis that tongxinluo capsule might exert its cardioprotective effect by preventing ventricular remodeling and improving coronary microvascular function in a rat model of doxorubicin-induced dilated cardiomyopathy (DCM). Rats that survived DCM induction were randomly divided into three groups to be given 1.5 g·kg^−1^·day^−1^ (TXL-H, *n* = 9) or 0.15 g·kg^−1^·day^−1^ (TXL-L, *n* = 10) of tongxinluo, or normal saline at the same volume (DCM-C, *n* = 10) intragastrically. Age matched normal rats treated with normal saline were used as normal controls (NOR-C, *n* = 9). After four weeks of treatment, the DCM-C, TXL-H, and TXL-L groups exhibited significant cardiac dysfunction, left ventricular remodeling, and coronary microvascular dysfunction, compared with the NOR-C rats. However, myocardial functional parameters were significantly improved and microvascular density (MVD) increased in the TXL-H group compared with the DCM-C group (all *P* < 0.01). Left ventricular remodeling was prevented. There were close linear relationships between CVF and LVEF (*r* = −0.683, *P* < 0.05), MVD and LVEF (*r* = 0.895, *P* < 0.05), and MVD and CVF (*r* = −0.798, *P* < 0.05). It was indicated that high-dose tongxinluo effectively improved cardiac function in rat model of DCM.

## 1. Introduction

Dilated cardiomyopathy (DCM) is characterized by ventricular chamber enlargement and systolic dysfunction with normal ventricular wall thickness [1]. It is an important cause of sudden cardiac death and heart failure and is the leading indication for cardiac transplantation in children and adults worldwide [2]. Doxorubicin (Dox) is an effective antitumor agent. Despite its use as a common chemotherapeutic agent, Dox use can also lead to cardiotoxicity. Multiple intravenous Dox treatments over a period of several months have been shown to induce cardiomyopathy and chronic heart failure in humans [3]. Study has also shown that rats treated with 15 mg/kg Dox develop DCM [4].

Although cardiac remodeling characterizes the natural history of DCM [5–7] and some drugs have been reported to have inhibitory effects on left ventricular remodeling [8], the mechanisms involved in the progressive deterioration of cardiac function are largely unknown. As dysfunction of the coronary microvessels is present from very early onset of the disease [9, 10], it has been proposed that impairment of coronary microcirculation may be responsible for the marked decrease in coronary flow reserve (CFR) [11] and may cause progressive contractile impairment, ventricular dilation, and heart failure [12]. It has been demonstrated that the presence and extent of early stage coronary microvascular dysfunction in DCM is an independent and relevant predictor of worse prognosis [13]. On the basis of these studies it is becoming evident that the coronary microcirculation is involved in the pathogenesis of DCM and should be considered as a new target of treatment in DCM [10, 12, 14].

Pharmaceutics of herbal medicine are undergoing rapid development in China. With the progression of modern technology, more and more herbal compound extracts are being authenticated, standardized, and administered successfully in clinical practice. Tongxinluo capsule is a compound preparation formulated on the meridian theory of traditional Chinese medicine and was officially approved for treatment of angina pectoris and ischemic stroke in 1996 by the State Food and Drug Administration of China [15, 16]. Pharmaceutical analysis has demonstrated that it contains multiple active components that may be responsible for multiple therapeutic effects, such as preventing ventricular remodeling [17, 18] and improving microvascular function [19, 20] in the brain and the heart. However, the result of these therapeutic effects on DCM has not been studied to date.

In the present study, we aimed at testing the hypothesis that tongxinluo might exert its cardioprotective effect by preventing ventricular remodeling and especially by improving coronary microvascular function in a rat model of doxorubicin-induced DCM.

## 2. Materials and Methods

### 2.1. Components and Fingerprint Chromatography of Tongxinluo Capsule

Tongxinluo capsule (authorized number: Z19980015, lot number: 101005) was provided by Shijiazhuang Yiling Pharmacy (Heibei, China). The herbal drugs were authenticated and standardized on marker compounds according to the Chinese Pharmacopoeia 2005 [21]. Tongxinluo contains 12 medicinal components (Table 1), which were ground to a superfine powder with diameters ≤ 10 *μ*m by a micronizer and prepared as capsules. To reduce dose variability of tongxinluo capsules among different batches, the species, origin, harvest time, medical parts, and concocted methods for each component were strictly standardized. Moreover, high performance liquid chromatography (HPLC) and gas chromatography (GC) for fingerprint analysis were applied to quantitate the components of the tongxinluo capsule [15, 22]. The detailed methods and results are described in the Supplementary Material available online at http://dx.doi.org/10.1155/2014/323870.

### 2.2. Animal Models

Doxorubicin-induced DCM was generated as described previously [23]. Briefly, fifty-four male Sprague-Dawley rats weighing 220 ± 10 g (Shanghai SLAC Laboratory Animal Co., Ltd.) were used in this study. After observation for one week in the normal laboratory environment on an ordinary diet, forty-five animals were administered doxorubicin hydrochloride (Zhejiang Haizheng Pharmaceutical Industry, China) intraperitoneally in six equal injections (each containing 2.5 mg/kg) over a period of 2 weeks for a total cumulative dose of 15 mg/kg. The remaining nine received the same volume of normal saline for the control. Two weeks after cessation of doxorubicin injection, both groups of rats were examined by transthoracic echocardiography to see whether the model was successful. All animals were treated humanely in accordance with the Principles of Laboratory Animal Care formulated by the Guide for the Care and Use of Laboratory Animal, published by the US National Institutes of Health. The protocol was approved by the Ethics Committee of Shanghai Chest Hospital Affiliated to Shanghai Jiao Tong University.

### 2.3. Grouping and Treatment

Sixteen (35.6%) of the 45 doxorubicin-administrated rats died from day 14 to 27. Animals that survived after DCM induction were randomly divided into three groups: (1) the high-dose tongxinluo group (TXL-H, *n* = 9): received tongxinluo superfine powder intragastrically at a dosage of 1.5 g·kg^−1^·day^−1^ for 4 weeks; (2) the low-dose tongxinluo group (TXL-L, *n* = 10): received tongxinluo superfine powder intragastrically at a dosage of 0.15 g·kg^−1^·day^−1^ for 4 weeks [24, 25]; (3) the DCM control group (DCM-C, *n* = 10): received normal saline intragastrically at the same volume as the tongxinluo-treated groups for 4 weeks. Age-matched normal rats treated with normal saline intragastrically were used as normal controls (NOR-C, *n* = 9). Throughout the treatment, the general appearance, behavior, body weight, and survival rate of the rats were observed every day.

### 2.4. Echocardiography Measurements

Before and after the treatment, transthoracic echocardiography was performed in all groups under chloral hydrate anesthesia in a supine position using a high-resolution small animal echocardiographic system (VisualSonics, Canada) equipped with a 7.5-MHz transducer. A two-dimensional short-axis view of the left ventricle was obtained at the level of the papillary muscle and two-dimensional targeted M-mode tracings were recorded. The detection index was as follows: left ventricular ejection fraction (LVEF), left ventricular end-systolic diameter (LVEDD), left ventricular end-systolic diameter (LVESD), and left ventricular fractional shortening (LVFS). All of the parameters were measured over 3 consecutive cardiac cycles.

### 2.5. Tissue Sample Preparation

After echocardiography measurement, hearts were arrested in diastole by intravenous injection of KCl (2 mol/L). The wet myocardium was isolated and weighed to calculate the ratio of heart weight (HW) to body weight (BW). The excised myocardium was sliced into three blocks (basal, mid-region, and apical) and the mid-region portion was fixed in 4% paraformaldehyde for 16–18 hours at 4°C followed by embedding in paraffin wax and cutting into 5 *μ*m slices for subsequent histologic and immunohistochemical analysis.

### 2.6. Histopathological Examination

Paraffin-embedded slices were stained with hematoxylin-eosin (HE) for morphologic examination or picrosirius red for interstitial fibrosis determination (collagen staining). For HE slices, myocyte diameter measurements were performed in 10 myocytes selected per field in 200-fold magnification by light microscopy (Leica, Germany) [26]. Short axis diameters of each myocyte were measured from the hearts of all groups of rats. Each average value was obtained from the data for 10 myocytes and was used as independent sampling data. In addition, myocyte hypertrophy, inflammatory cell infiltrations, vacuolization, and the extent of vascular hyperemia were observed in HE sections. For collagen staining, An Axioplan II KS 400 microscope (Carl Zeiss, Germany) was used to capture at least 4 randomly selected images from each slide using the ×10 objective. Collagen fibers were stained red or green under linearly polarized light, and the area of myocardial fibrosis in left ventricular tissue sections stained with picrosirius red was quantified using a color image analyzer (CIA-102, Olympus, Tokyo, Japan). The result was presented as interstitial collagen volume fraction (CVF) which was calculated as the sum of all connective tissue area divided by the sum of all connective tissue and cardiac myocyte areas, as demonstrated previously in a number of studies [27–29].

### 2.7. Immunohistochemistry

To detect the microvessels in the myocardium, endothelial cells were stained with the specific primary antibody to CD31 (1 : 50, ab28364, Abcam, Cambridge, UK), according to the product specification. Microvascular density (MVD) in the myocardium was determined by a double-headed light microscope (Leica, Germany) using the counting method introduced by Weidner [30]. Briefly, the myocardial area in rat specimens containing the maximum number of microvessels was identified by scanning at low power (×40 and ×100). Individual microvessels were then counted at ×400 magnification, where one field is equivalent to 0.09 mm^2^. Each positive endothelial cell cluster of immunoreactivity in contact with the selected field was counted as an individual vessel in addition to the morphologically identifiable vessels with a lumen. The number of countable microvessels in five fields was recorded, and the average value was defined as MVD for the case. Assessment of MVD was done without knowledge of any pathological data and was performed by two technicians in our microscopy laboratory. Disagreements on what constituted a microvessel were resolved by consensus.

### 2.8. Statistical Analysis

Values are presented as mean ± standard deviation (SD) for continuous variables and absolute number (percentage) for categorical variables. Numerical data were analyzed using One-Way analysis of variance (ANOVA) followed by least significant difference (LSD) as post-hoc, whereas categorical data were compared using a chi-square test. Kapla-Meier analysis was used to obtain survival curves. The Log-Rank test for pairwise over strata was used to compare the survival rates among groups, and the Pearson correlation coefficient, to assess the correlation between any two variables of CVF, MVD, and LVEF. Statistical analysis was performed using SPSS 13.0 statistical software (SPSS Inc., Chicago, Illinois, USA). A 2-tailed *P* value of less than 0.05 was considered statistically significant.

## 3. Results

### 3.1. Tongxinluo Increased Survival Rates in Rats with DCM

**Figure 1:**
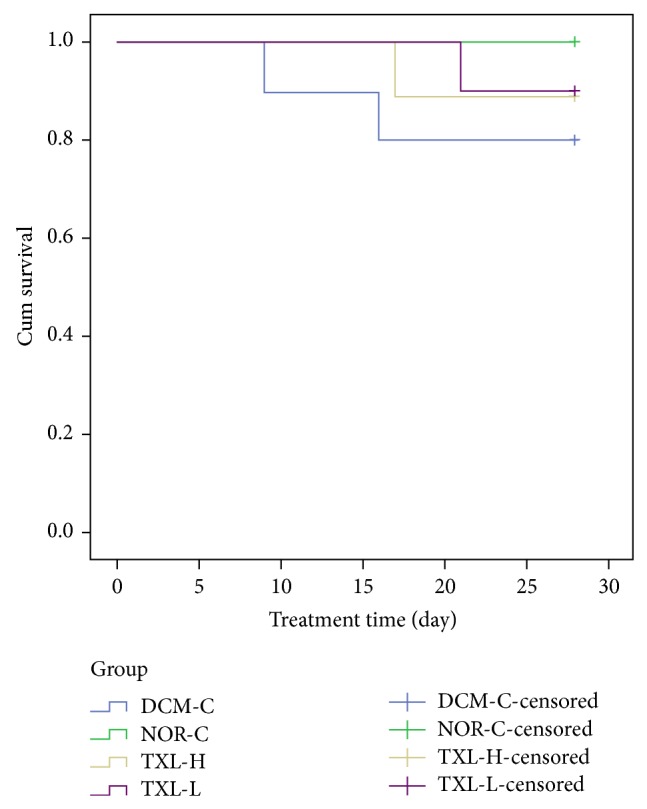
Effects of tongxinluo on cumulative survival rate. TXL-H: high-dose tongxinluo group; TXL-L: low-dose tongxinluo group; DCM-C: DCM control group; NOR-C: normal control group.

The survival study started after the DCM model was successfully set up, and after completion of the tongxinluo treatment, cumulative survival rate was 88.9% (8/9), 90.0% (9/10), 80.0% (8/10), and 100.0% (9/9) in the TXL-H, TXL-L, DCM-C, and NOR-C groups, respectively. Although rats in the tongxinluo-treated groups showed a higher survival rate than that in the DCM-C group, no statistically significant differences were revealed among these three groups (all *P* > 0.05, Figure 1). The rats died presumably from severe congestive heart failure, malignant arrhythmias, or refractory ascites, as reported previously in a number of studies [31].

### 3.2. High-Dose Tongxinluo Decreased Heart Weight and Ratio of Heart Weight to Body Weight in Rats with DCM

**Figure 2:**
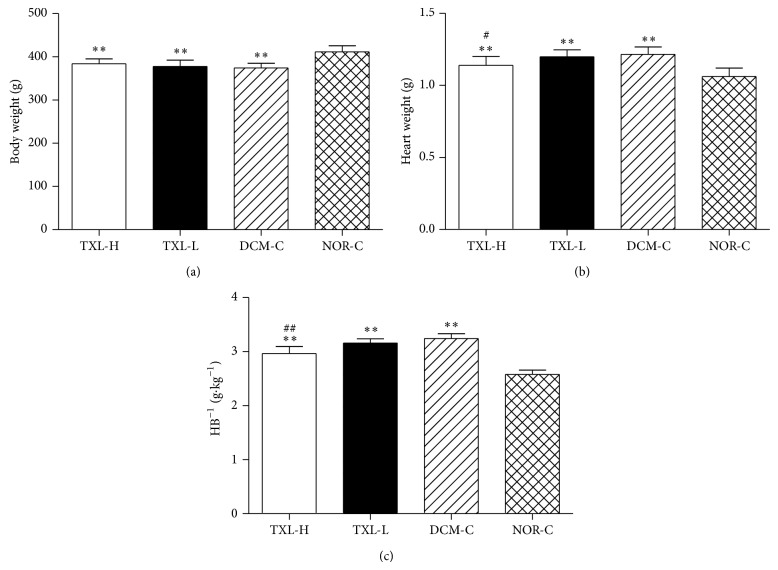
Effects of tongxinluo on body weight (a), heart weight (b), and ratio of heart weight to body weight (H B^−1^) (c). Values are mean ± SD. ^**^ *P* < 0.01 versus the NOR-C group. ^#^ *P* < 0.05 and ^##^ *P* < 0.01 versus the DCM-C group.

The body weight (BW), heart weight (HW), and ratio of heart weight to body weight (H B^−1^) are shown in Figure 2. The BW was significantly larger in the NOR-C group than that in the TXL-H, TXL-L, and DCM-C groups (411.64 ± 13.86 g versus 383.89 ± 11.35 g, 377.50 ± 14.72 g, 374.53 ± 10.73 g, respectively, all *P* < 0.01), respectively, but it did not differ among the latter three groups (all *P* > 0.05). The HW and H B^−1^ were significantly larger in the DCM-C group (1.22 ± 0.05 g and 3.24 ± 0.09 g·kg^−1^) than those in the NOR-C group (1.06 ± 0.06 g and 2.58 ± 0.08 g·kg^−1^, both *P* < 0.01). The HW and H B^−1^ were significantly decreased in the TXL-H group (1.14 ± 0.06 g and 2.97 ± 0.13 g·kg^−1^) compared to the DCM-C group (1.22 ± 0.05 g and 3.24 ± 0.09 g·kg^−1^, both *P* < 0.05). No statistically significant differences were shown in HW and H B^−1^ between the TXL-L group (1.20 ± 0.05 g and 3.16 ± 0.08 g·kg^−1^) and the DCM-C group (1.22 ± 0.05 g and 3.24 ± 0.09 g·kg^−1^, both *P* > 0.05).

### 3.3. High-Dose Tongxinluo Improved Cardiac Function in Rats with DCM

**Figure 3:**
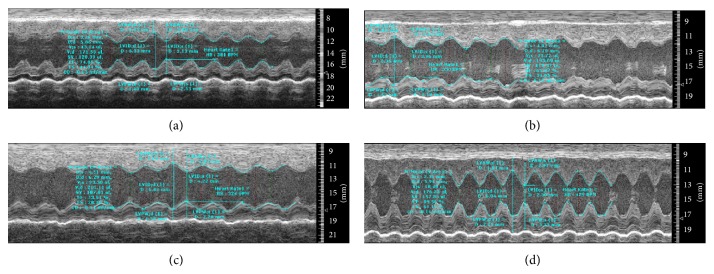
Representative echocardiographic views of a left ventricle from each group. (a) the TXL-H group; (b) the TXL-L group; (c) the DCM-C group; (d) the NOR-C group.

Figure 3 shows a representative echocardiography view of hearts from each group after treatment. As shown in Table 2, echocardiography analyses before treatment indicated that, compared with those in the NOR-C group, rats in the DCM-C, TXL-H, and TXL-L groups exhibited significant left ventricular dilation and systolic dysfunction, as evidenced by significantly increased LVEDD and LVESD (both *P* < 0.01), and significantly decreased LVEF and LVFS (both *P* < 0.01). However, the LVESD, LVEF, and LVFS in the TXL-H group were significantly improved after treatment when compared with the DCM-C group (all *P* < 0.01), while no significant difference in the four values was shown between the TXL-L group and DCM-C group (all *P* > 0.05), demonstrating that high-dose tongxinluo treatment could markedly ameliorate cardiac dysfunction.

### 3.4. High-Dose Tongxinluo Improved Left Ventricle Remodeling in Rats with DCM

**Figure 4:**
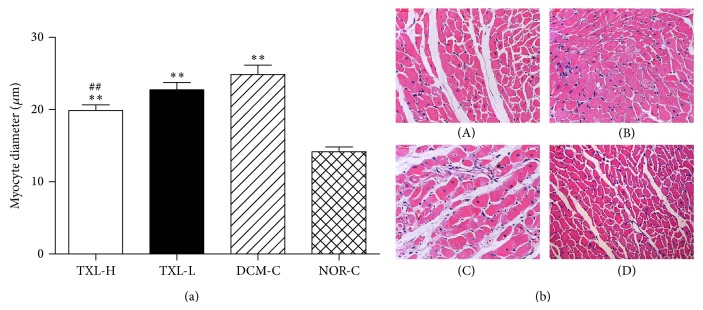
The effect of tongxinluo on myocardial morphology. (a) Quantitative analysis of myocyte diameter of the cross-sectional tissue slices of hearts. Values are mean ± SD. ^**^ *P* < 0.01 versus the NOR-C group. ^##^ *P* < 0.01 versus the DCM-C group. (b) Representative HE staining images (×200). ((b)-(A)) the TXL-H group; a little of vacuolar degeneration and myocyte hypertrophy. ((b)-(B)) the TXL-L group; myocyte hypertrophy, vacuolar degeneration, and inflammation cells infiltration. ((b)-(C)) The DCM-C group; obvious myocyte hypertrophy, numerous vacuolar degeneration and local vascular hyperemia. ((b)-(D)) the NOR-C group; the normal heart.

Cardiac tissue sections were examined using HE staining for morphological analysis. The myocyte size in the DCM-C group was significantly larger than that in the NOR-C group (24.86 ± 3.69 versus 14.15 ± 2.04, *P* < 0.01). However, the TXL-H group had significantly reduced myocyte size when compared with the DCM-C group (19.89 ± 2.20 versus 24.86 ± 3.69, *P* < 0.01), while there was no significant difference between the TXL-L group and DCM-C group (22.75 ± 3.00 versus 24.86 ± 3.69, *P* > 0.05) (Figure 4(a)). The representative HE staining images (×200) were shown in Figure 4(b). Obvious cardiac hypertrophy, vacuolar degeneration and local vascular hyperemia were detected in the hearts of the DCM-C group. However, the extent of pathological changes in the myocardial samples from the TXL-H group was less severe than that in the DCM-C group.

**Figure 5:**
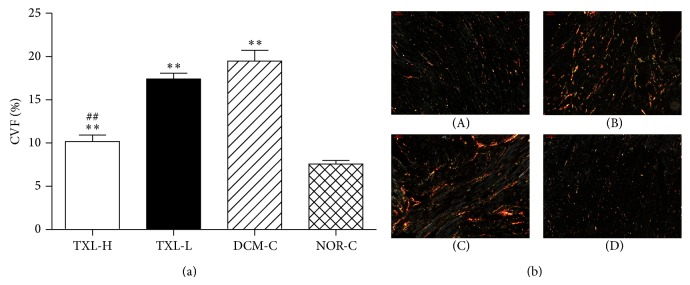
The effect of tongxinluo on left ventricular interstitial fibrosis. (a) Quantification of picrosirius red staining after treatment. Values are mean ± SD. CVF, collagen volume fraction. ^**^ *P* < 0.01 versus the NOR-C group. ^##^ *P* < 0.01 versus the DCM-C group. (b) Representative images using picrosirius red staining from each group at ×10 objective magnification. ((b)-(A)) the TXL-H group; ((b)-(B)) the TXL-L group; ((b)-(C)) the DCM-C group; ((b)-(D)) the NOR-C group.

Figure 5 illustrates the quantification of picrosirius red staining (Figure 5(a)) and shows representative images of left ventricular interstitial fibrosis using picrosirius red staining from each group (Figure 5(b)). Specifically, quantitative analysis revealed a significantly higher extent of left ventricular interstitial fibrosis in the DCM-C group when compared with the NOR-C group (19.47 ± 3.56 versus 7.57 ± 1.24, *P* < 0.01). However, compared with the DCM-C group, high-dose tongxinluo administration significantly alleviated fibrotic deposits in the left ventricular wall (10.18 ± 2.09 versus 19.47 ± 3.56, *P* < 0.01), while the TXL-L group showed a tendency to lower CVF (17.40 ± 2.01 versus 19.47 ± 3.56, *P* > 0.05).

### 3.5. High-Dose Tongxinluo Increased Microvascular Density in Rats with DCM

**Figure 6:**
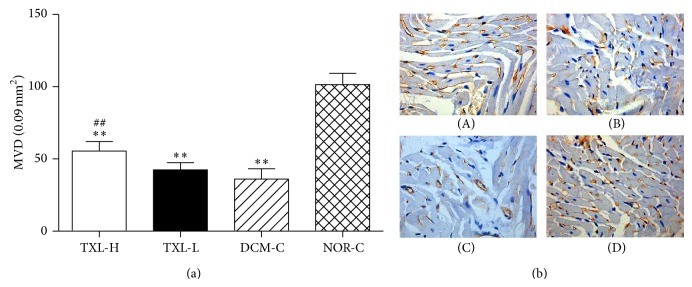
The effect of tongxinluo on angiogenesis. (a) Quantitative analysis of microvessel counts after treatment. Values are mean ± SD. MVD, microvascular density. ^**^ *P* < 0.01 versus the NOR-C group. ^##^ *P* < 0.01 versus the DCM-C group. (b) Representative CD31 staining images (×400). ((b)-(A)) the TXL-H group; ((b)-(B)) the TXL-L group; ((b)-(C)) the DCM-C group; ((b)-(D)) the NOR-C group.

The values for microvessel counts and the representative images in each group after treatment are given in Figure 6. Overall, when compared with that of the NOR-C group, the MVD in myocardium was significantly decreased in the TXL-H, TXL-L, and DCM-C groups (101.44 ± 7.81 versus 55.38 ± 6.74, 42.44 ± 5.03, 36.00 ± 7.15, respectively; all *P* < 0.01), respectively. However, the MVD was significantly increased in the TXL-H group compared to the DCM-C group (55.38 ± 6.74 versus 36.00 ± 7.15, *P* < 0.01) while no significant difference was found between the TXL-L group and DCM-C group (42.44 ± 5.03 versus 36.00 ± 7.15, *P* > 0.05), which demonstrated the dose-dependent angiogenesis-promoting effect of tongxinluo.

### 3.6. The Correlations between CVF and LVEF, and CVF and LVEF Were Negative While MVD and LVEF Were Positively Correlated

**Figure 7:**
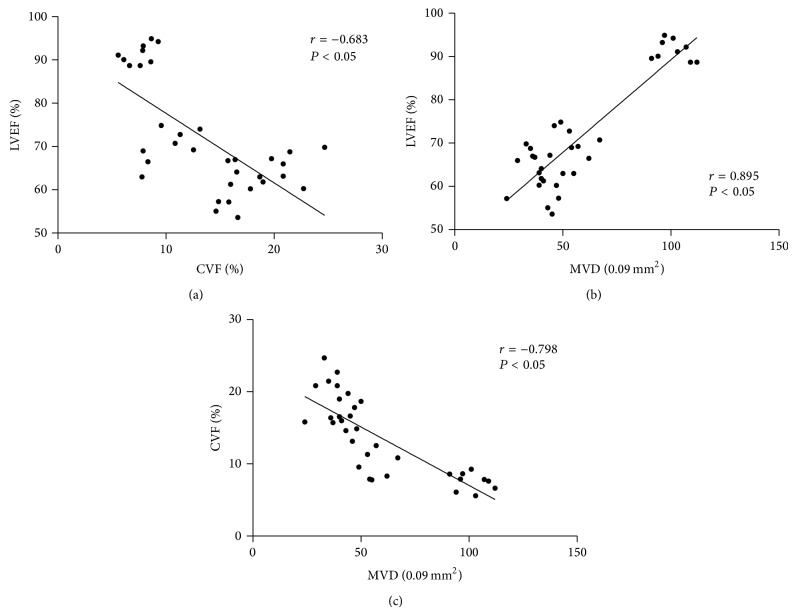
Relationships between collagen volume fraction (CVF), microvascular density (MVD), and left ventricular ejection fraction (LVEF). (a) Correlation between CVF and LVEF; (b) correlation between MVD and LVEF; (c) correlation between MVD and CVF. CVF, collagen volume fraction; MVD, microvascular density; LVEF, left ventricular ejection fraction.

Correlations between any two variables of CVF, MVD, and LVEF are shown in Figure 7. Overall, there were significant negative correlations between CVF and LVEF (*r* = −0.683, *P* < 0.05), and MVD and CVF (*r* = −0.798, *P* < 0.05), respectively. A significant positive correlation was also observed between MVD and LVEF (*r* = 0.895, *P* < 0.05).

## 4. Discussion

In the present study, using a rat model of dilated cardiomyopathy induced by doxorubicin hydrochloride in our laboratory, we examined the beneficial effects of tongxinluo on progression of cardiac dysfunction with dilated cardiomyopathy. We found that high-dose tongxinluo (1.5 g·kg^−1^·day^−1^) attenuated left ventricular remodeling and promoted angiogenesis more effectively than low-dose tongxinluo (0.15 g·kg^−1^·day^−1^).

The tongxinluo capsule is an officially approved treatment in China for angina pectoris and ischemic stroke and the compound preparation was formulated on the basis of the meridian theory of traditional Chinese medicine [15, 16]. The routine clinical dose range of tongxinluo capsule (3 capsules, 3 times per day) approximates to 60 mg·kg^−1^·day^−1^ of tongxinluo powder, which may correspond to the dose of 400 mg·kg^−1^·day^−1^ in rats by taking account of the ratio of 6.3 [25]. In published experimental studies in rats, the highest dose effective for cardiac dysfunction was 1.5 g·kg^−1^·day^−1^ [24]. Thus, the higher dose of 1.5 g·kg^−1^·day^−1^ used in our study corresponds to 225 mg·kg^−1^·day^−1^ of tongxinluo powder, a dose approximately four times as high as the routine dose of 60 mg·kg^−1^·day^−1^ for patients. The lower dose of 0.15 g·kg^−1^·day^−1^ in our study corresponds to 22.5 mg·kg^−1^·day^−1^ of tongxinluo powder, similar to one third of the routine clinical dose.

To our knowledge, ventricular remodeling is characterized by both quantitative and qualitative alterations of cardiac extracellular matrix and hypertrophy of cardiomyocytes [17]. Some basic studies have demonstrated that tongxinluo could inhibit ventricular remodeling in animals with spontaneous hypertension or myocardial infarction [17, 18, 32]. In our study, using two different doses, we found that tongxinluo capsule dose-dependently reduced the myocyte size in rats with DCM. In addition, the collagen volume fraction, an important index of cardiac fibrosis, was significantly lower in the TXL-H group than the DCM-C group, and histopathologic examinations showed that the degree of fibrosis was obviously lower when treated with high-dose tongxinluo. These effects of tongxinluo were associated with the prevention of ventricular remodeling. In a review of the literature, the authors have reported that ventricular remodeling could importantly affect the function of the ventricle [33], and Montera et al. [34] demonstrated a significant negative correlation between CVF and LVEF with correlation coefficient of 0.7, which is a little more than that in our study (*r* = −0.683, *P* < 0.05). Therefore, the ventricular remodeling-preventing effects of tongxinluo may contribute to the improvement of cardiac function in rats with DCM.

More recent studies have shown that the coronary microcirculation may be directly affected in cardiomyopathies. Abnormalities in coronary flow reserve are often observed in patients with DCM [35–38]. Similar findings commonly suggest the presence of coronary artery stenosis, which will not be confirmed at cardiac catheterization in these patients, but abnormalities at the coronary microvascular level might similarly be responsible for these observations. Structural alternations of the coronary microcirculation, known as coronary remodeling, including incomplete arteriolar wall [39], as well as decreased microvessel density [14, 39, 40], provide an anatomical basis for a decreased coronary flow reserve in DCM. Furthermore, Tsagalou et al. [11] observed a close linear relationship between CFR and MVD in patients with idiopathic dilated cardiomyopathy (*r* = 0.756, *P* = 0.0001), suggesting the important role of MVD in the decreased CFR and even the progression of cardiac dysfunction. Similarly, the impairment of MVD in the myocardium was also observed in our study. However, high-dose tongxinluo administration significantly increased MVD in the myocardium, demonstrating the angiogenesis-promoting effect of tongxinluo. As evidenced by the strong correlation between MVD and LVEF (*r* = 0.895, *P* < 0.05), this tongxinluo effect may also contribute to the improvement of cardiac function in rats with DCM.

Furthermore, a number of studies have suggested that changes of microvascular dysfunction play an important role in the development of ventricular remodeling. Particularly, the possibility was raised that microvascular dysfunction in the myocardium may be a powerful and independent predictor contributing to the remodeling of left ventricle [41–43]. This has been emphasized in a study demonstrating that the improvement of microvascular function early after myocardial infarction is beneficial in preventing left ventricular remodeling [42]. Also supporting our findings of microvascular dysfunction, MVD and CVF have a strong relationship (*r* = −0.798, *P* < 0.05), and left ventricular remodeling attenuates beneficially after promoting angiogenesis by treatment with high-dose tongxinluo.

However, the specific mechanisms of the angiogenesis-promoting effects of tongxinluo in our study are not clear but may be implied from previous studies. In a model of focal cerebral ischemia, Chang et al. [19] demonstrated that tongxinluo could significantly improve the MVD of ischemic stroke rats, which may be related to the increase of NO and VEGF expression. Hu et al. [44] found that tongxinluo could induce angiogenesis in bone marrow mesenchymal stem cells (MSCs), and the underlying mechanisms are associated with increased migration ability of MSCs and the upregulation of MMP-2 and VEGF expressions. All of the above mechanisms of tongxinluo may contribute to the angiogenesis of the left ventricle of the rats with DCM in this investigation, although the exact mechanism needs further studies.

There are several limitations to this study. First, four weeks' administration of tongxinluo is a little short and that may be why a significant difference in LVEDD between the TXL-H group and DCM-C group was not observed, though the TXL-H group showed an obvious tendency to lower LVEDD. Second, the components of tongxinluo capsule were clear and pharmaceutical analysis has demonstrated that peoniflorin, ginsenoside Rg1, ginsenoside Rb1, jujuboside A, and jujuboside B are the five most important active components in the tongxinluo capsule. However, it is not known which component is the most efficacious one responsible for the observed protective effect on cardiac dysfunction of the rats with DCM. We need to further fractionate the individual components of the compound and evaluate the protective effect of each individual component and their interactions. Third, although the beneficial effect of tongxinluo on cardiac function has been confirmed in our study, the detailed molecular mechanisms by which tongxinluo prevents ventricular remodeling and promotes angiogenesis require further investigation. Finally, human dilated cardiomyopathy is thought to have a variety of causes. Therefore, the clinical courses and pathological findings in this cardiomyopathy are not uniform. Thus, the present results only provide some small insight into the effectiveness of tongxinluo treatment for dilated cardiomyopathy.

## 5. Conclusion

In conclusion, the present study indicates that administration of high-dose tongxinluo effectively improves cardiac function in a rat model of doxorubicin-induced DCM, which may be attributed to prevention of left ventricular remodeling and, more particularly, promotion of angiogenesis by tongxinluo.

## Supplementary Material

Tongxinluo contains 12 medicinal components which were ground to a superfine powder and prepared as capsules. High performance liquid chromatography (HPLC) and gas chromatography (GC) for fingerprint analysis were applied to quantitate the components of the tongxinluo capsule. The Supplementary Material describes the detailed methods and results of HPLC and GC for fingerprint analysis.

## Figures and Tables

**Table 1:** Formulation of tongxinluo capsule.

Components	Voucher specimen number	Part used	Amount (%)
*Panax ginseng* C.A. Mey. (extraction)	11,001	Root and rhizome	1.68
*Paeonia lactiflora* Pall. (extraction)	11,003	Root	1.56
*Ziziphus jujuba* Mill. Var. *spinosa *(Bunge) Hu ex H.F.Chou (extraction)	11,002	Seed	1.17
*Santalum album *L. (extraction)	11,004	Heartwood of stem	0.35
*Dalbergia odorifera* T.C.Chen (extraction)	11,005	Heartwood of stem and root	4.00
*Steleophaga plancyi* (Boleny) (Microoryzae farina)	12,003	Female dried body	18.11
*Scolopendra subspinipes mutilans* L. Koch (farina)	12,001	Dried body	3.62
*Hirudo nipponica* Whitman (farina)	12,004	Dried body	27.33
*Cryptotympana pustulata* Fabricius (farina)	12,005	Skin	18.11
*Buthus martensii* Karsch (farina)	12,002	Dried body	18.11
*Boswellia carteri* (farina)	11,006	Resin	5.93
*Borneolum syntheticum* (artificial)	11,007	C_10_H_18_0	3.62

**Table 2:** Echocardiography results in each group before and after treatment.

Groups		LVEDD	LVESD	LVEF	LVFS
TXL-H	(b)	6.71 ± 0.27^**^	3.77 ± 0.31^**^	65.93 ± 5.17^**^	39.62 ± 4.34^**^
	(a)	6.59 ± 0.26^**^	3.57 ± 0.37^∗∗,##^	70.00 ± 3.98^∗∗,##^	41.98 ± 3.43^∗∗,##^

TXL-L	(b)	6.66 ± 0.25^**^	3.80 ± 0.33^**^	66.69 ± 3.04^**^	39.94 ± 1.80^**^
	(a)	6.82 ± 0.18^**^	3.90 ± 0.21^**^	63.32 ± 3.37^**^	37.89 ± 2.77^**^

DCM-C	(b)	6.70 ± 0.38^**^	3.82 ± 0.37^**^	62.79 ± 6.07^**^	36.34 ± 5.05^**^
	(a)	6.85 ± 0.40^**^	4.06 ± 0.34	61.57 ± 6.17	35.16 ± 5.06

NOR-C	(b)	6.05 ± 0.26	2.00 ± 0.30	90.88 ± 1.66	64.90 ± 4.00
	(a)	6.15 ± 0.24	2.04 ± 0.31	91.40 ± 2.35	64.80 ± 4.62

LVEDD, left ventricular end-diastolic diameter; LVESD, left ventricular end-systolic diameter; LVEF, left ventricular ejection fraction; LVFS, left ventricular fractional shortening. ^**^ *P* < 0.01 versus the NOR-C group. ^##^ *P* < 0.01 versus the DCM-C group. b: before treatment; a: after treatment.
