# Supplementary material for: Traditional Chinese Medicine Tongxinluo Improves Cardiac Function of Rats with Dilated Cardiomyopathy
Source: Evid Based Complement Alternat Med. 2014 Dec 28;2014:323870. doi: 10.1155/2014/323870 (PMC4295346; doi:10.1155/2014/323870)
Supplement: Supplementary file 1 — Tongxinluo contains 12 medicinal components which were ground to a superfine powder and prepared as capsules. High performance liquid chromatography (HPLC) and gas chromatography (GC) for fingerprint analysis were applied to quantitate the components of the tongxinluo capsule. The Supplementary Material describes the detailed methods and results of HPLC and GC for fingerprint analysis. [file 323870.f1.doc]

**Supplementary materials**

**Traditional Chinese medicine tongxinluo improves cardiac function of rats with dilated cardiomyopathy**

**Supplementary material Legend:**

Name of Traditional Chinese Medicine: tongxinluo capsule

Authorized number: Z19980015

Lot Number: 101005

**Methods**

1. The HPLC fingerprint:

The HPLC system was equipped with an Agilent HP-1100 HPLC instrument. Separation was performed on a Waters Symmetry C-18 (4.6mm×250mm, 5μm) analytical column with mobile phase consisting of acetonitrile and water with gradient elution at a flow rate of 1.0 ml/min and the column temperature at 30°C. The UV wavelength used for detection was set at 203 nm and the analysis time was 90min. Thirteen common peaks of tongxinluo were identified on the HPLC fingerprints．

1. The GC fingerprint:

GC analyses were carried out on an Agilent 6890N gas chromatograph equipped with a flame ionization detector. Separation was performed on an Agilent DB –624 (30.0m×535μm×3.0μm) capillary column with nitrogen used as a carrier gas and a split ratio of 10: 1. The injector and detector temperature were set at 200 and 250°C, respectively. The column temperature was programmed isothermally at 50°C for 2 min, and then increased first at a rate of 10°C /min to 160°C and subsequently at a rate of 5°C /min to 200°C, where it was held for 5 min of isothermal operation. Eight common peaks on the GC fingerprints of the tongxinluo capsule were identified．

**Supplementary Figure 1 The HPLC fingerprint of tongxinluo capsule**


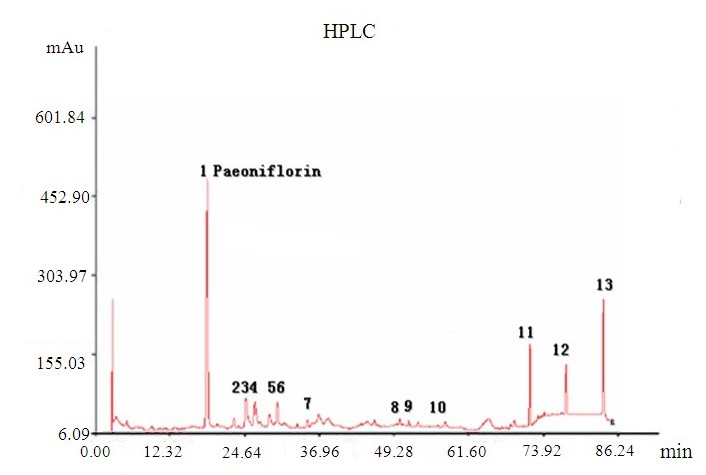


The major components of HPLC fingerprint:

Peak 1: paeoniflorin

Peak 7: ginsenoside Rg_1_

Peak 8: ginsenoside Rb_1_

Peak 9: jujuboside A

Peak 10: jujuboside B

**Supplementary Figure 2** **The GC fingerprint of tongxinluo capsule**


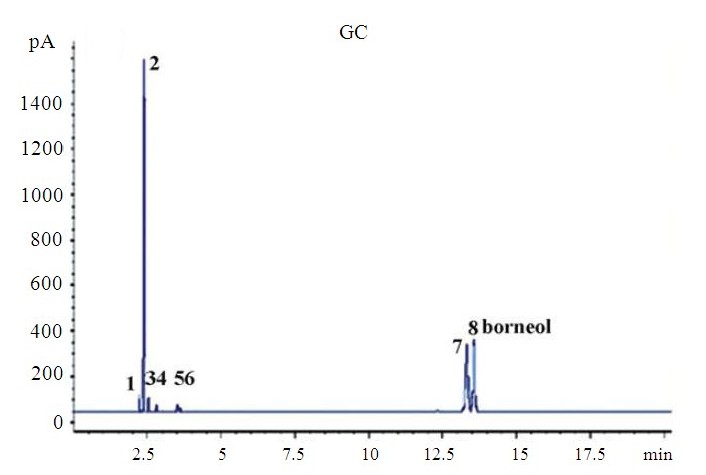


The major components of GC fingerprint:

Peak 7: isoborneol

Peak 8: borneol
